# Access systems in general practice: a systematic scoping review

**DOI:** 10.3399/BJGP.2023.0149

**Published:** 2024-09-03

**Authors:** Abi Eccles, Carol Bryce, Annelieke Driessen, Catherine Pope, Jennifer MacLellan, Toto Gronlund, Brian D Nicholson, Sue Ziebland, Helen Atherton

**Affiliations:** Warwick Applied Health, Warwick Medical School, University of Warwick, Coventry, UK.; Warwick Applied Health, Warwick Medical School, University of Warwick, Coventry, UK.; Nuffield Department of Primary Care Health Sciences, University of Oxford, Oxford, UK; post-doctoral researcher, Anthropology Department, University of Amsterdam, Amsterdam, the Netherlands.; Nuffield Department of Primary Care Health Sciences, University of Oxford, Oxford.; Nuffield Department of Primary Care Health Sciences, University of Oxford, Oxford.; Primary Care, Population Sciences and Medical Education, University of Southampton, Southampton, UK.; Nuffield Department of Primary Care Health Sciences, University of Oxford, Oxford.; Nuffield Department of Primary Care Health Sciences, University of Oxford, Oxford.; Primary Care, Population Sciences and Medical Education, University of Southampton, Southampton, UK.

**Keywords:** appointments and schedules, general practice, primary health care

## Abstract

**Background:**

Access to GP appointments is increasingly challenging in many high-income countries, with an overstretched workforce and rising demand. Various access systems have been developed and evaluated internationally.

**Aim:**

To systematically consolidate the current international evidence base related to different types of GP access systems.

**Design and setting:**

Scoping review examining international literature.

**Method:**

Literature searches were run across relevant databases in May 2022. Title, abstract, and full-text screenings were carried out. Data from included studies were extracted and mapped to synthesise the components and aims within different GP access systems.

**Results:**

In total, 49 studies were included in the review. The majority of these were set in the UK. Some access systems featured heavily in the literature, such as Advanced Access, telephone triage, and online consultations, and others less so. There were two key strategies adopted by systems that related to either changing appointment capacity or modifying patient pathways. Components related to these strategies are summarised and illustrated as a schematic representation. Most rationales behind access systems were practice, rather than patient, focused. ‘Add-on’ systems and aims for efficiency have become more popular in recent years.

**Conclusion:**

This synthesis provides a useful tool in understanding access systems’ aims, design, and implementation. With focus on alleviating demand, patient-focused outcomes appear to be underinvestigated and potentially overlooked during design and implementation. More recently, digital services have been promoted as offering patient choice and convenience. But a context where demand outweighs resources challenges the premise that extending choice is possible.

## Introduction

Access to general practice is a prominent, often contentious concern for policymakers, politicians, service providers, and the public. Governments use it as a high-profile benchmark for health service performance.^1^^,^^2^ Access comprises key elements including choice, timeliness, the physical aspects of access, and financial considerations in countries where GP care is not free at the point of use.^2^ In many countries, policy focuses on speed and convenience,^3^ despite evidence that continuity of care is both safer^4^^,^^5^ and preferred by patients.^6^ Recent amendments to the UK’s General Medical Services contract legislate that GP services must progress enquiries (for example, offer appointments or signpost to appropriate services) on the same day that patients make contact.^7^ However, the GPC England (General Practitioners Committee, the representative body for GPs in England) asserts general practices currently do not have the workforce or resources to adhere to delivering this.^8^

Access becomes a problem when demand exceeds supply, a reality increasingly facing health systems globally. Unmet demand for primary care has risen in recent years,^9^^–^11 fuelled by retention and recruitment crises in general practice,^11^^–^13 an ageing and increasingly multimorbid population, and changes to care delivery in response to the COVID-19 pandemic.^14^ Patient-reported satisfaction with access to UK general practice has reduced year on year since 2018 in the UK.^15^

General practice access systems have mainly focused on managing ‘supply and demand’ by the use of patient triage assessment by phone or online,^16^ by varying appointment availability, length or number of problems considered,^17^ diverting to other staff such as physician associates,^18^ or offering other modalities, such as asynchronous consultations online via text message/email, real-time telephone, or video consultations.^19^^,^^20^ A recent systematic review noted a paucity of evidence about the impact of remote consultations on continuity of care, and suggested that multiple, interrelated factors influence continuity and the quality of access.^21^ Digital technologies often offer solutions to the access problem^22^ but there is evidence of their unintended consequences.^23^^,^^24^

Research on GP access systems has sought to inform service delivery^20^^,^^25^^–^27 but often focuses on a single aspect (for example, digital platforms) or initiative (for example, Advanced Access). The intention of this scoping review was to map the current evidence base relating to access systems to inform research and guide decisions in general practice.

**Table:** How this fits in

Access to GP appointments poses challenges for general practice and frequently gains media attention. Various booking systems have been adopted to overcome issues; however, there is a lack of evidence consolidating understanding of such approaches. This systematic scoping review provides a broad lens that summarises and maps the different types of GP access systems that have been studied and the rationales behind them. It provides a comprehensive overview to aid understanding, while highlighting gaps that appear to be overlooked in the literature.

## Method

The aim of this study was to describe the different types of access systems previously studied and reported in the research literature in the past 20 years, thus conducting a scoping review was appropriate.^28^ Using established scoping review methods,^29^ five steps were followed:

1. identifying the research question;
2. identifying relevant studies;
3. study selection;
4. charting the data; and
5. collating, summarising, and reporting the results.

This scoping review was conducted as part of a larger funded project looking at access to general practice.^30^ A scoping review design was chosen to provide a rapid summary of research conducted in the previous 20 years to inform the next stage of the project and map the research field. A 20-year cut off was applied to ensure that the research studies identified were as contemporary as possible while including some of the history of research in this field.

### Identifying the research question

To identify and describe the different types of access systems studied the authors developed the research question: What types of access systems for general practice have been empirically studied?

### Identifying relevant studies

Studies that examined the use, application, or evaluation of an access system within a general practice setting were included. Access systems were defined as those providing access to an appointment for a consultation. The focus was on routine general practice care excluding studies investigating access to ‘out-of-hours’ urgent care services, even if in primary care settings. Participants of interest were patients, staff, or both. Studies focusing on access limited to a specific condition or follow-up appointments were excluded.

Studies with any empirical study design (quantitative, qualitative, or mixed methods) published in English were included. Editorials, debate pieces, conference abstracts, and reviews were excluded.

The search strategy was limited to studies published after January 2001 (to ensure contemporary relevance). On 24 May 2022 the search was run within MEDLINE; Embase; PsycINFO; Cochrane Trials; Web of Science; and Scopus databases (see Supplementary Box S1).

### Study selection

Titles, abstracts, and full texts were independently screened by two researchers before selection for the review. A third reviewer resolved any disagreements about inclusions.

### Charting the data

Study characteristics and information about the access system studied were extracted. This included access systems’ descriptions; components; rationales; modes of contact; and staff members facilitating use. In line with guidance for scoping reviews^31^ and the study aims, a critical appraisal of the studies was not undertaken; however, the relevance of each included study (that is, does the research address the topic and allow the authors to add to the descriptions of access systems) and its credibility (that is, does the research support the conclusions drawn) was assessed. This approach has been successfully used in similar reviews.^32^^,^^33^

### Collating, summarising, and reporting results

The included studies were summarised using narrative synthesis,^34^ this involved three steps:

1. preliminary synthesis using text and tables to present the studies’ characteristics and describe the access systems;
2. further synthesis of study characteristics and access systems descriptions to develop an initial overview and schematic representation. This detailed the key elements within studies and visually mapped out organisational approaches and pathways used within access systems; and
3. refinement of synthesis with input from key stakeholders (including GPs and the public) to assess robustness and applicability. This allowed further development of the synthesis and the schematic representation, illustrating how different access systems fit within general practices.

## Results

### Search results

**Figure 1.**
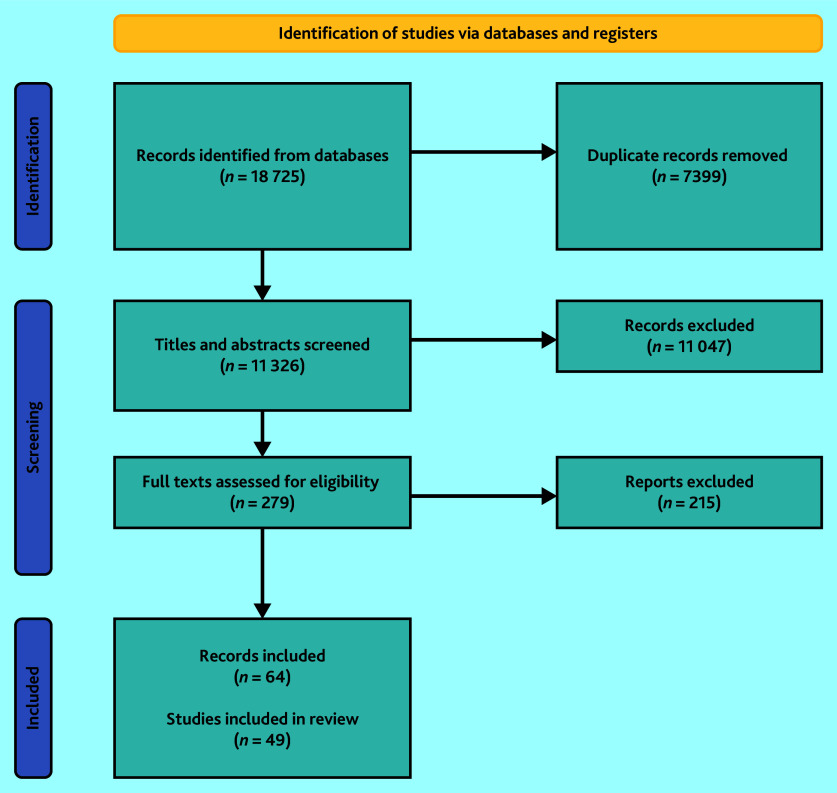
Flow diagram: results from the screening process.

The initial search yielded 11 326 (deduplicated) records. After screening titles and abstracts, 279 full texts were assessed. Of these, 49 studies^25^^,^^35^^–^78^,^^94^^–^97 reported across 64 publications,^25^^,^^35^^–^97 were included (Figure 1). Most included studies were UK based (*n* = 33),^25^^,^^35^^,^^37^^,^^40^^–^43^,^^45^^–^47^,^^52^^,^^54^^,^^55^^,^^57^^,^^58^^,^^60^^–^74^,^^77^^,^^94^^,^^96^ followed by Sweden (*n* = 4),^48^^,^^49^^,^^59^^,^^75^ Norway (*n* = 3),^39^^,^^78^^,^^97^ Spain (*n* = 3),^50^^,^^53^^,^^95^ Australia (*n* = 2),^51^^,^^56^ Denmark (*n* = 2),^36^^,^^38^ New Zealand (*n* = 1),^76^ and the Netherlands (*n* = 1).^44^ Of the included studies, 20 were quantitative,^39^^,^^44^^,^^50^^,^^52^^,^^54^^,^^58^^,^^60^^–^63^,^^67^^,^^69^^–^73^,^^76^^,^^78^^,^^95^^,^^96^ 17 were qualitative, 35^,^^36^^,^^41^^,^^42^^,^^48^^,^^49^^,^^51^^,^^53^^,^^56^^,^^59^^,^^64^^–^66^,^^68^^,^^77^^,^^94^^,^^97^ and 12 used mixed methods.^25^^,^^37^^,^^38^^,^^40^^,^^43^^,^^45^^–^47^,^^55^^,^^57^^,^^74^^,^^75^ Levels of relevance varied between studies, but all provided partial or comprehensive evidence that informed the review.

### Impact of COVID-19

No studies set out to examine the impact of the COVID-19 pandemic, but two were unexpectedly affected by it.^55^^,^^76^ Ure in 2022 reported that increased respiratory complaints led to adaptations to the access system, whereby nurses (rather than GPs) were deployed to triage patients.^76^ Following then current New Zealand health guidelines, those with respiratory complaints were offered an in-person review that led to fewer patients being treated remotely. Jones *et al* reported no access system adaptations in their UK study but reported that social distancing led to increased use of the online platform being studied.^55^

### The access systems

Some approaches featured prominently in the literature. Advanced Access, different forms of telephone triage, and online consultation platforms were the subject of several studies and are outlined in the first part of Box 1. Other studies examined bespoke approaches to access, often with similar components to those mentioned above. These approaches included redirection; appointments with other (non-GP) healthcare professionals; direct booking; and introducing new appointment types, timings, or modes of access. Components of these systems are outlined in the latter part of Box 1. There is repetition within Box 1 as access systems had elements in common. Some access systems were ‘whole systems’ that all patients used, others were ‘add-ons’ introduced alongside existing systems, and, in some cases, this was not clear from the publication. ‘Whole systems’ have been studied consistently since 2001, whereas ‘add-ons’ became more popular from 2017 onwards, reflecting the advent of ‘add-on’ digital alternatives for contacting GPs.^41^^,^^43^^,^^46^^,^^47^^,^^55^^,^^78^^,^^80^

**Box 1.** The access systems

**Access system**	**Presence in literature**
**Approaches commonly studied**	

Advanced access	Advanced Access dominated the literature, with nine studies examining this approach^35^^,^^38^^,^^45^^,^^52^^,^^56^^,^^66^^,^^69^^,^^93^^,^^94^ and three reporting variations of it.^61^^,^^67^^,^^77^ These studies spanned across three countries: the UK, Australia, and Denmark. Advanced Access aims to manage demand, often by offering same-day appointments to prevent long waiting times. When setting up the system the pattern of appointment requests is assessed, and capacity temporarily adjusted to clear any backlog. Appointment access focuses on seeing patients on the day they contact the surgery and limiting how far ahead patients can book appointments. Patients are often allowed to address >1 health concern per visit, and typically practices reorganise staffing levels to clear backlog and/or provide contingency staffing.^56^
Telephone triage	Eleven studies referred to a form of telephone triage,^40^^,^^42^^,^^52^^,^^54^^,^^57^^,^^60^^,^^62^^,^^65^^,^^71^^,^^72^^,^^76^ whereby patients discussed their problem over the phone with a member of staff in the first instance, with subsequent advice or appointments based on this interaction. This approach aims to improve access, alleviate demand for face-to-face appointments, and reduce non- attendance. Five of these studies examined telephone triage by GPs,^52^^,^^54^^,^^57^^,^^60^^,^^65^ three investigated telephone triage by nurses,^42^^,^^71^^,^^72^ two looked at triage by both GPs and nurses,^40^^,^^76^ and one did not state which staff member carried out the triage.^62^
Online consultation platforms	Seven studies assessed online consultations or e-consultations whereby patients submit an online form describing their request or problem.^25^^,^^41^^,^^43^^,^^46^^,^^47^^,^^55^^,^^95^ A staff member assesses the content and responds, fulfilling an administrative request, or arranging a consultation (via phone, online messaging, or face to face). This approach has also been described as ‘online triage’. Six of these studies were UK based^25^^,^^41^^,^^43^^,^^46^^,^^47^^,^^55^ and one was Spanish.95 Online consultations are often used alongside other access systems, providing an alternative mode of contact for patients. They are often designed to encourage self-management.

**Other approaches studied**	
Redirection	Redirection was a common approach in which patients were signposted to self-help advice, NHS 111 (UK), pharmacies, or to make solely administrative requests, for example, repeat prescriptions
Non-GP healthcare professional appointments	Some systems introduced more appointments with non-GPs. Instead of seeing a GP in the first instance, patients were triaged to an appointment with another professional instead, for example, a practice nurse, psychotherapist, or counsellor.^59^^,^^68^^,^^70^^,^^72^^,^^96^
Direct booking	Two studies examined systems that enable direct booking. That is, patients have a choice of GP appointment slots to select and book directly without gatekeeping or triage. Those studies that did examine direct booking were assessing the introduction of a new ‘add-on’ mode for direct booking. These included direct booking via an online platform^53^ and SMS text messaging.^64^
Limiting appointment availability	Having a limit on the number of appointments that were pre-bookable or the number of same-day appointments^51^^,^^77^ attempts to manage high demand.
New appointment types or timings	Some systems introduced new types or timings of appointments, such as extended hours outside the working day^96^ and short review appointments for those with long-term conditions.^74^
New modes of access	For most patients, initial contact to book a GP appointment was via telephone. However, some systems introduced new modes of access including online,^48^^,^^49^^,^^57^^,^^78^^,^^97^ SMS,^39^^,^^64^ and an in-person ‘sit-and-wait’ surgery.^67^

*SMS = short message service.*

### Rationale for different access systems

Nine studies did not report the access system’s rationale.^41^^,^^46^^,^^58^^,^^61^^,^^64^^,^^66^^,^^69^^,^^76^^,^^77^ Where reported, access systems were most commonly intended to manage demand and improve efficiency (*n* = 28),^35^^–^40^,^^42^^–^44^,^^47^^–^51^,^^53^^,^^54^^,^^56^^,^^60^^,^^62^^,^^65^^,^^71^^,^^72^^,^^74^^,^^75^^,^^78^^,^^95^^–^97 with ‘efficiency’ more commonly reported from 2017 onwards. Some described aims related to improvements for patients, such as convenience, reduced waiting time, and access to healthcare advice (*n* = 17),^25^^,^^37^^–^40^,^^45^^,^^47^^,^^52^^,^^57^^,^^59^^,^^63^^,^^65^^,^^67^^,^^70^^,^^74^^,^^78^^,^^94^ often coupled with practice-focused aims such as efficiency or managing demand (*n* = 8/17 studies).^37^^–^40^,^^47^^,^^65^^,^^74^^,^^78^ Two UK-based studies referred to government policy when describing the reasons behind introduction of the access system.^68^^,^^77^ Studies examining online platforms in particular stated aims to improve efficiency and reduce face-to-face visits (see Supplementary Box S2). Only six studies examined how the access systems were used by specific groups;^37^^,^^40^^,^^53^^,^^65^^,^^76^^,^^78^ however, these aspects were not the primary focus of these studies.

### Categorising access systems

As noted above, the rationale behind most access systems was described as being to alleviate pressure on general practice, often specifically to reduce GP workload. The approaches could broadly be distinguished into two groups as those designed to:

- modify patients’ pathways to obtaining appointments (includes any type of consultation, for example, call back from, or asynchronous messaging, with healthcare professionals); or
- alter appointment capacity (through reorganisation of appointments).

Systems were designed according to one of these approaches or they combined both of these approaches.

#### Modifying patients’ pathways

Approaches to modify appointment booking pathways varied. These included: triage of patients based on need before offering an appointment; changing the mode of contact; arranging appointments with non-GP healthcare professionals; or signposting to self-help advice and other services (NHS 111 or pharmacist). How these pathways were administered differed, some systems used staff judgement whereas others used algorithms (either automated or as a guide for staff members to follow).

#### Altering appointment capacity

Many systems revised the availability and organisation of bookable appointments. Strategies included limiting how far in advance appointments could be booked; introducing a ‘sit-and-wait’ open surgery; deploying one GP to see urgent same-day patients; and introducing different types of appointments (described above). Some systems offered new appointment types with aims to have more efficient use of services, for example, extended hours to alleviate the pressure during normal opening hours, and shorter routine appointments for patients with long-term conditions. Change to appointment capacity was sometimes adopted alongside modifications to booking pathways.

**Figure 2.**
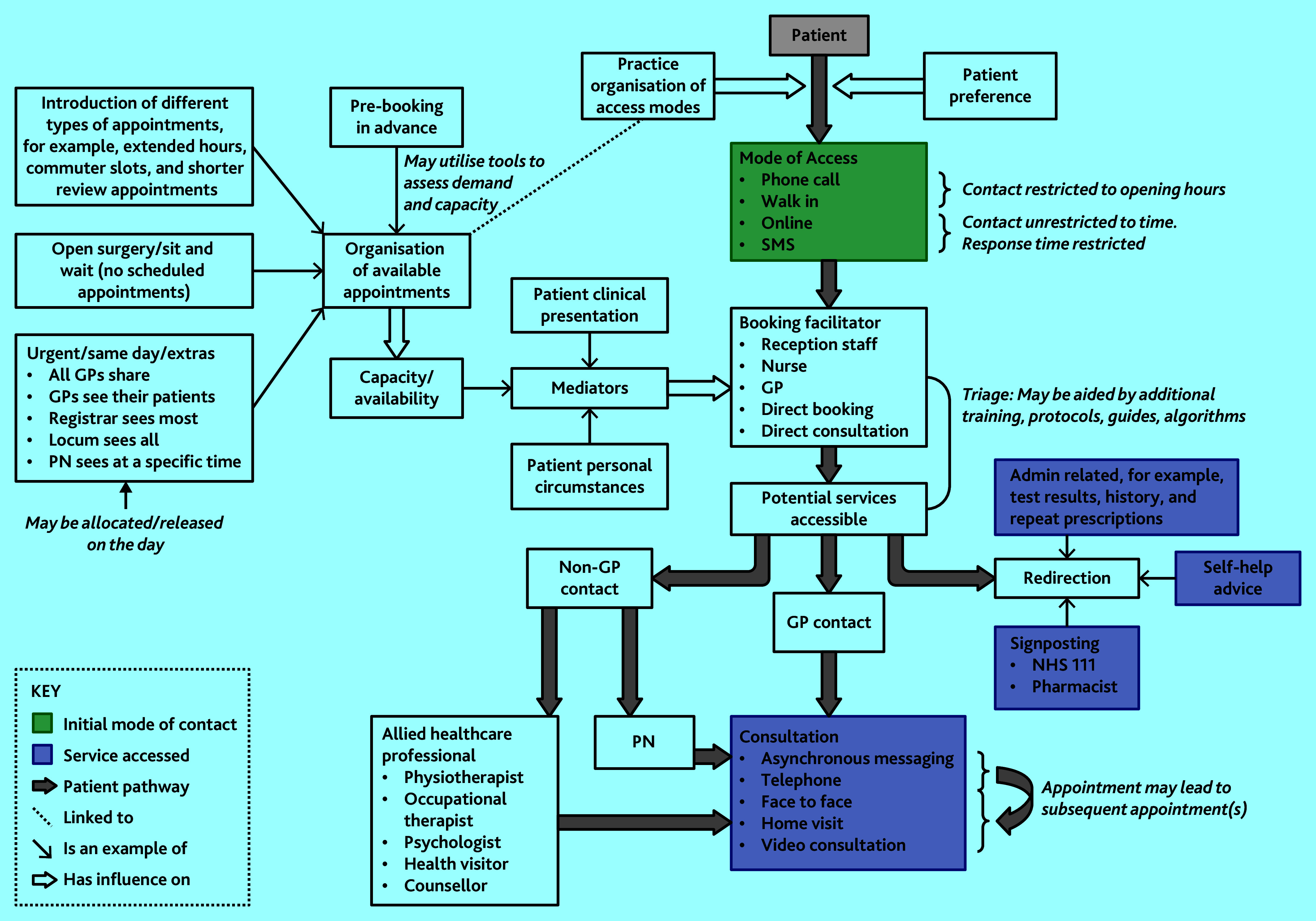
Schematic representation of the components of access systems empirically studied. PN = practice nurse. SMS = short message service.

Included studies examined access systems that adopted one, or both, of these strategies. The relationship between these access strategies is complex and dynamic, as illustrated in the schematic representation (Figure 2), which maps the key components, how they influence each other, and connections between them.

## Discussion

### Summary

This study has described the varied and dynamic components that make up GP access systems reported in the literature and offers a schematic representation of the findings (Figure 2). The figure summarises and distinguishes the different approaches to GP access and offers a tool to identify gaps in the evidence base. Reflecting changes in government policy, and innovation (and promotion) of digital approaches, the review has identified imbalance in the type of systems studied, with some featuring more frequently in the literature and with some changes of reported rationale over time. Managing demand was a common and consistent aim within access system research, with efficiency aims featuring more in recent years, as did the ‘add-on’ approaches associated with newer digital access systems.

### Strengths and limitations

This international scoping review allowed comprehensive consolidation of evidence from research conducted since 2001 about approaches to GP access systems set in countries with universal health care, although only including studies published in English was a limitation of the review. This scoping review did not intend to map the entire history of access research, as this was beyond its scope and would be better suited to a systematic review design. Including studies published since 2001 enabled this scoping review to provide an overview of systems that are used by, and relevant to, current general practice settings. Over the past 20 years general practice has seen much change in how access is organised and delivered alongside societal level changes to communication technologies, including the introduction of broadband internet in the early 2000s^98^ and growing widespread use of increasingly sophisticated smartphones by the general public since 2007.^99^^,^^100^ The study’s inclusion period captures these important changes, but this scoping review does not include studies pre-dating 2001 and may have omitted useful content as a result.

Most of the studies included were set in the UK, reflecting a strong field of academic primary care research and the importance of general practice within the NHS. This makes the findings particularly relevant and applicable to current day British general practice and the challenges of access, while being potentially transferable elsewhere. All the studies included were assessed as credible and relevant, strengthening the review’s findings.

Consultation with stakeholders highlighted some access systems and adaptations not (yet) present in the literature, for example, more recent digital approaches. Thus, this scoping review is not an exhaustive account of all existing/emerging systems and adaptations that are currently in use, rather it comprehensively reflects published research. The schematic representation may provide a useful visual aid for policymakers, politicians, service providers, and the public/patients when considering and discussing the components of access systems and how these interact.

### Comparison with existing literature

In recent years digital services have been promoted as offering patient choice and convenience, with an expectation that UK general practices will offer 25% of their appointments as bookable directly online.^101^ However, the rhetoric of choice and convenience contrasts with those access systems that aim to alleviate demand by limiting appointment availability. Such conflicting priorities may — to some extent — explain why patient satisfaction in the UK is at an all-time low.^15^ A context where demand outweighs resources challenges the premise that extending choice is possible, without significantly more GP resource. Even if giving patients a choice of appointment slots does not lead to increased pressures, non-attendance, or inappropriate use,^102^ it is easy to appreciate why service providers are wary that it might. A modelling study that examined the introduction of digital approaches to access examined supply-related demand and forecasted increases in workload.^103^

The system known as ‘Advanced Access’ featured heavily in the literature, which may be because it was highlighted as an effective model in the UK Government’s £48 million to ‘Primary Care Access Fund’ between 2002 and 2003.^104^ This coincided with ‘The NHS Plan’ access targets to provide all primary care patients an appointment within 24 h, and a GP appointment within 48 h.^105^ Such targets may help explain why Advanced Access systems incorporate ‘same-day appointments’ to manage access, although it should be noted that Advanced Access does not necessarily stipulate ‘same-day appointments’.^94^ Such adaptations demonstrate how access systems are shaped by context and political factors. Advanced Access is framed politically as providing prompt access for patients, while at the practice level it is used to manage demand. However, limits to bookable appointments may lead to unmet needs for some patients that go undetected within the studies that typically focus on GP workload as their outcome measure. Although prompt access to appointments is arguably appealing, the potential impact on access and continuity of care for those patients unable to prebook for a different day, or with specific GPs, ought to be considered with the introduction of such systems. Recognition of patients unable to prebook appointments potentially led to the recent changes to the UK’s General Medical Services contract, which stipulate GP services must progress enquiries on the same day that patients make contact,^7^ but the practicalities and impact of this is yet to be seen.

### Implications for research and practice

Most access systems that have been the subject of research are intended to manage demand, often by reducing pressure on GP workload, rather than seeking to improve patient-focused outcomes such as ease of access, satisfaction, health status, and safety. With emphasis on organisation-focused outcomes, more patient-related outcomes appear to have been, at times, overlooked in the design of research studies about access systems. Movement towards aims for efficiency in recent years suggests there is recognition that the solution is more nuanced than reducing resource pressure but may instead lie in patients accessing the most appropriate appointment for them.

In the UK, equality legislation stipulates that all public sector entities should remove or minimise disadvantages to those with protected characteristics,^106^ which include age, disability, gender reassignment, race, religion or belief, sex, and sexual orientation. Yet the authors of the current study found that very few studies examined how access systems worked for such groups, and investigations were limited in those that did; a gap that was highlighted by the current study’s patient and public advisory panel in response to the initial scoping review draft. This evidence gap has political and policy implications, and the current study’s wider review findings suggest that research and policy may need to look beyond managing demand and GP workload to find practice- and patient-focused solutions. Research into implementation of such systems, how they work within the dynamics of practices, the associated costs, their sustainability, and the potential impact for different groups would be of value.

The published literature tells us little about how these systems have adapted or were shaped by their contexts, for example, changes to GP contracts, political environments, and unexpected challenges (such as COVID-19). Informed by this scoping review, the authors have begun a major ethnographic study (the GP SUS study, funded by the National Institute for Health and Care Research Health and Social Care Delivery Research Programme) to examine how pre-existing GP access systems were used, adapted, or abandoned according to local contexts, as well as the COVID-19 pandemic and its aftermath.

## Data

All cited articles are publicly available.

## References

[1] Cowling TE, Gunning EJ (2016). Access to general practice in England: political, theoretical, and empirical considerations. Br J Gen Pract.

[2] Boyle S, Appleby J, Harrison A (2010). A rapid view of access to care.

[3] Cowling TE, Harris MJ, Majeed A (2015). Evidence and rhetoric about access to UK primary care. BMJ.

[4] Pereira Gray DJ, Sidaway-Lee K, White E (2018). Continuity of care with doctors — a matter of life and death? A systematic review of continuity of care and mortality. BMJ Open.

[5] Sandvik H, Hetlevik Ø, Blinkenberg J, Hunskaar S (2022). Continuity in general practice as predictor of mortality, acute hospitalisation, and use of out-of-hours care: a registry-based observational study in Norway. Br J Gen Pract.

[6] Gerard K, Salisbury C, Street D (2008). Is fast access to general practice all that should matter? A discrete choice experiment of patients’ preferences. J Health Serv Res Policy.

[7] Government HM (2023). The National Health Service (General Medical Services Contracts and Personal Medical Services Agreements) (Amendment) (No. 2) Regulations 2023. https://www.legislation.gov.uk/uksi/2023/449/schedule/1/made.

[8] British Medical Association (2024). GP access: meeting the reasonable needs of patients. https://www.bma.org.uk/advice-and-support/gp-practices/gp-service-provision/gp-access-meeting-the-reasonable-needs-of-patients.

[9] Morken T, Rebnord IK, Maartmann-Moe K, Hunskaar S (2019). Workload in Norwegian general practice 2018 – an observational study. BMC Health Serv Res.

[10] Crosbie B, O’Callaghan ME, O’Flanagan S (2020). A real-time measurement of general practice workload in the Republic of Ireland: a prospective study. Br J Gen Pract.

[11] (2022). Royal Australian College of General Practitioners. General practice health of the nation 2022.

[12] Kjosavik SR (2018). Ongoing recruitment crisis In Norwegian general practice. Scand J Prim Health Care.

[13] Owen K, Hopkins T, Shortland T, Dale J (2019). GP retention in the UK: a worsening crisis. Findings from a cross-sectional survey. BMJ Open.

[14] Wanat M, Hoste M, Gobat N (2021). Transformation of primary care during the COVID-19 pandemic: experiences of healthcare professionals in eight European countries. Br J Gen Pract.

[15] Dawson M, Rehsi A (2023). 2023 GP Patient Survey Results released. https://www.ipsos.com/en-uk/2023-gp-patient-survey-results-released.

[16] Lake R, Georgiou A, Li J (2017). The quality, safety and governance of telephone triage and advice services – an overview of evidence from systematic reviews. BMC Health Serv Res.

[17] Sampson R, O’Rourke J, Hendry R (2013). Sharing control of appointment length with patients in general practice: a qualitative study. Br J Gen Pract.

[18] Nelson P, Martindale A-M, McBride A (2018). Skill-mix change and the general practice workforce challenge. Br J Gen Pract.

[19] McKinstry B, Walker J, Campbell C (2002). Telephone consultations to manage requests for same-day appointments: a randomised controlled trial in two practices. Br J Gen Pract.

[20] Hammersley V, Donaghy E, Parker R (2019). Comparing the content and quality of video, telephone, and face-to-face consultations: a non-randomised, quasi-experimental, exploratory study in UK primary care. Br J Gen Pract.

[21] Ladds E, Khan M, Moore L (2023). The impact of remote care approaches on continuity in primary care: a mixed-studies systematic review. Br J Gen Pract.

[22] Paddison C, McGill I (2022). Digital primary care: improving access for all?.

[23] Turner A, Morris R, Rakhra D (2022). Unintended consequences of online consultations: a qualitative study in UK primary care. Br J Gen Pract.

[24] Ziebland S, Hyde E, Powell J (2021). Power, paradox and pessimism: on the unintended consequences of digital health technologies in primary care. Soc Sci Med.

[25] Carter M, Fletcher E, Sansom A (2018). Feasibility, acceptability and effectiveness of an online alternative to face-to-face consultation in general practice: a mixed-methods study of webGP in six Devon practices. BMJ Open.

[26] Huibers L, Moth G, Carlsen AH (2016). Telephone triage by GPs in out-of-hours primary care in Denmark: a prospective observational study of efficiency and relevance. Br J Gen Pract.

[27] Grønning A (2021). Struggling with and mastering e-mail consultations: a study of access, interaction, and participation in a digital health care system. Nordicom Rev.

[28] Munn Z, Peters MDJ, Stern C (2018). Systematic review or scoping review? Guidance for authors when choosing between a systematic or scoping review approach. BMC Med Res Methodol.

[29] Arksey H, O’Malley L (2005). Scoping studies: towards a methodological framework. Int J Soc Res Methodol.

[30] Nuffield Department of Primary Care Health Sciences Access to General Practice: Innovation, impact and sustainable change (GP-SUS). https://www.phc.ox.ac.uk/research/health-experiences/gp-sus.

[31] Tricco AC, Lillie E, Zarin W (2018). PRISMA extension for scoping reviews (PRISMA-ScR): checklist and explanation. Ann Intern Med.

[32] Ziebland S, Wyke S (2012). Health and illness in a connected world: how might sharing experiences on the internet affect people’s health?. Milbank Q.

[33] Atherton H, Ziebland S (2016). What do we need to consider when planning, implementing and researching the use of alternatives to face-to-face consultations in primary healthcare?. Digit Health.

[34] Popay J, Roberts H, Sowden A (2006). Guidance on the conduct of narrative synthesis in systematic reviews. https://www.lancaster.ac.uk/media/lancaster-university/content-assets/documents/fhm/dhr/chir/NSsynthesisguidanceVersion1-April2006.pdf.

[35] Ahluwalia S, Offredy M (2005). A qualitative study of the impact of the implementation of advanced access in primary healthcare on the working lives of general practice staff. BMC Fam Pract.

[36] Andersen RS, Aarhus R (2019). Reconfiguring diagnostic work in Danish general practice; regulation, triage and the secretaries as diagnostician. Anthropol Med.

[37] Atherton H, Brant H, Ziebland S (2018). The potential of alternatives to face-to-face consultation in general practice, and the impact on different patient groups: a mixed-methods case study.

[38] Bang M, Pedersen HS, Bech BH (2020). Advanced Access scheduling in general practice and use of primary care: a Danish population-based matched cohort study. BJGP Open.

[39] Bergmo TS, Kummervold PE, Gammon D, Dahl LB (2005). Electronic patient-provider communication: will it offset office visits and telephone consultations in primary care?. Int J Med Inform.

[40] Campbell JL, Fletcher E, Britten N (2015). The clinical effectiveness and cost-effectiveness of telephone triage for managing same-day consultation requests in general practice: a cluster randomised controlled trial comparing general practitioner-led and nurse-led management systems with usual care (the ESTEEM trial). Health Technol Assess.

[41] Casey M, Shaw S, Swinglehurst D (2017). Experiences with online consultation systems in primary care: case study of one early adopter site. Br J Gen Pract.

[42] Charles-Jones H, May C, Latimer J, Roland M (2003). Telephone triage by nurses in primary care: what is it for and what are the consequences likely to be?. J Health Serv Res Policy.

[43] Cowie J, Calveley E, Bowers G, Bowers J (2018). Evaluation of a digital consultation and self-care advice tool in primary care: a multi-methods study. Int J Environ Res Public Health.

[44] de Groot RA, de Haan J, Bosveld HEP (2002). The implementation of a call-back system reduces the doctor’s workload, and improves accessibility by telephone in general practice. Fam Pract.

[45] Dixon S, Sampson FC, O’Cathain A, Pickin M (2006). Advanced access: more than just GP waiting times?. Fam Pract.

[46] Eccles A, Hopper M, Turk A, Atherton H (2019). Patient use of an online triage platform: a mixed-methods retrospective exploration in UK primary care. Br J Gen Pract.

[47] Edwards HB, Marques E, Hollingworth W (2017). Use of a primary care online consultation system, by whom, when and why: evaluation of a pilot observational study in 36 general practices in South West England. BMJ Open.

[48] Eldh AC, Sverker A, Bendtsen P, Nilsson E (2020). Health care professionals’ experience of a digital tool for patient exchange, anamnesis, and triage in primary care: qualitative study. JMIR Hum Factors.

[49] Entezarjou A, Bolmsjo BB, Calling S (2020). Experiences of digital communication with automated patient interviews and asynchronous chat in Swedish primary care: a qualitative study. BMJ Open.

[50] Fabrellas N, Vidal A, Amat G (2011). Nurse management of ‘same day’ consultation for patients with minor illnesses: results of an extended programme in primary care in Catalonia. J Adv Nursing.

[51] Garth B, Temple-Smith M, Clark M (2013). Managing same day appointments–a qualitative study in Australian general practice. Aust Fam Physician.

[52] Gill N, Freeman G (2007). Continuity of care and rapid access: the potential impact of appointment systems. Prim Health Care Res Dev.

[53] Gonzalez F, Cimadevila B, Garcia-Comesana J (2018). Telephone consultation in primary care. J Health Organ Manag.

[54] Jiwa M, Mathers N, Campbell M (2002). The effect of GP telephone triage on numbers seeking same-day appointments. Br J Gen Pract.

[55] Jones RB, Tredinnick-Rowe J, Baines R (2022). Use and usability of GP online services: a mixed-methods sequential study, before and during the COVID-19 pandemic, based on qualitative interviews, analysis of routine eConsult usage and feedback data, and assessment of GP websites in Devon and Cornwall, England. BMJ Open.

[56] Knight AW, Padgett J, George B, Datoo MR (2005). Reduced waiting times for the GP: two examples of “advanced access” in Australia. Med J Aust.

[57] Lawless M, Wright E, Davidson J (2016). A collaborative approach to improving patient access in general practice: impact of three different pilot schemes in 12 general practices in Greenwich. London J Prim Care.

[58] Luthra M, Marshall MN (2001). How do general practices manage requests from patients for ‘same-day’ appointments? A questionnaire survey. Br J Gen Pract.

[59] Maun A, Engström M, Frantz A (2014). Effective teamwork in primary healthcare through a structured patient-sorting system -a qualitative study on staff members’ conceptions. BMC Fam Pract.

[60] McKinstry B, Walker J, Campbell C (2002). Telephone consultations to manage requests for same-day appointments: a randomised controlled trial in two practices. Br J Gen Pract.

[61] Meade JG, Brown JS (2006). Improving access for patients - a practice manager questionnaire. BMC Fam Pract.

[62] Miller D, Loftus AM, O’Boyle PJ (2019). Impact of a telephone-first consultation system in general practice. Postgrad Med J.

[63] Morgan CL, Beerstecher HJ (2011). Satisfaction, demand, and opening hours in primary care: an observational study. Br J Gen Pract.

[64] Neville RG, Reed C, Boswell B (2008). Early experience of the use of short message service (SMS) technology in routine clinical care. Inform Prim Care.

[65] Newbould J, Abel G, Ball S (2017). Evaluation of telephone first approach to demand management in English general practice: observational study. BMJ.

[66] Offredy M, Ahluwalia S (2006). Ensuring successful implementation of advanced access in primary care: a qualitative study. Clin Manag.

[67] Pascoe SW, Neal RD, Allgar VL (2004). Open-access versus bookable appointment systems: survey of patients attending appointments with general practitioners. Br J Gen Pract.

[68] Perry C, Thurston M, Killey M, Miller J (2005). The nurse practitioner in primary care: alleviating problems of access?. Br J Nurs.

[69] Pickin M, O’Cathain A, Sampson FC, Dixon S (2004). Evaluation of advanced access in the national primary care collaborative. Br J Gen Pract.

[70] Pritchard A, Kendrick D (2001). Practice nurse and health visitor management of acute minor illness in a general practice. J Adv Nursing.

[71] Richards DA, Godfrey L, Tawfik J (2004). NHS Direct versus general practice based triage for same day appointments in primary care: cluster randomised controlled trial. BMJ.

[72] Richards DA, Meakins J, Tawfik J (2002). Nurse telephone triage for same day appointments in general practice: multiple interrupted time series trial of effect on workload and costs. BMJ.

[73] Salisbury C, Montgomery AA, Simons L (2007). Impact of advanced access on access, workload, and continuity: controlled before- and-after and simulated-patient study. Br J Gen Pract.

[74] Slater J, Malik S, Davey P, Grant S (2021). Improving access to primary care: a mixed-methods approach studying a new review appointment system in a Scottish GP practice. BMJ Open Qual.

[75] Thorn J, Maun A, Bornhoft L (2010). Increased access rate to a primary health-care centre by introducing a structured patient sorting system developed to make the most efficient use of the personnel: a pilot study. Health Serv Manage Res.

[76] Ure A (2022). Investigating the effectiveness of virtual treatment via telephone triage in a New Zealand general practice. J Prim Health Care.

[77] Windridge K, Tarrant C, Freeman GK (2004). Problems with a ‘target’ approach to access in primary care: a qualitative study. Br J Gen Pract.

[78] Zanaboni P, Fagerlund AJ (2020). Patients’ use and experiences with e-consultation and other digital health services with their general practitioner in Norway: results from an online survey. BMJ Open.

[79] Ball SL, Newbould J, Corbett J (2018). Qualitative study of patient views on a ‘telephone-first’ approach in general practice in England: speaking to the GP by telephone before making face-to-face appointments. BMJ Open.

[80] Banks J, Farr M, Salisbury C (2018). Use of an electronic consultation system in primary care: a qualitative interview study. Br J Gen Pract.

[81] Campbell JL, Fletcher E, Britten N (2014). Telephone triage for management of same-day consultation requests in general practice (the ESTEEM trial): a cluster-randomised controlled trial and cost-consequence analysis. Lancet.

[82] Farr M, Banks J, Edwards HB (2018). Implementing online consultations in primary care: a mixed-method evaluation extending normalisation process theory through service co-production. BMJ Open.

[83] Goodall S, Montgomery A, Banks J (2006). Implementation of Advanced Access in general practice: postal survey of practices. Br J Gen Pract.

[84] Holt TA, Fletcher E, Warren F (2016). Telephone triage systems in UK general practice: analysis of consultation duration during the index day in a pragmatic randomised controlled trial. Br J Gen Pract.

[85] Murdoch J, Varley A, Fletcher E (2015). Implementing telephone triage in general practice: a process evaluation of a cluster randomised controlled trial. BMC Fam Pract.

[86] Newbould J, Exley J, Ball S (2019). GPs’ and practice staff’s views of a telephone first approach to demand management: a qualitative study in primary care. Br J Gen Pract.

[87] Pickin M, O’Cathain A, Sampson F (2010). The impact of advanced access on antibiotic prescribing: a controlled before and after study. Fam Pract.

[88] Richards DA, Meakins J, Godfrey L (2004). Survey of the impact of nurse telephone triage on general practitioner activity. Br J Gen Pract.

[89] Richards DA, Meakins J, Tawfik J (2004). Quality monitoring of nurse telephone triage: pilot study. J Adv Nurs.

[90] Sampson F, Pickin M, O’Cathain A (2008). Impact of same-day appointments on patients satisfactions with general practice appointment systems. Br J Gen Pract.

[91] Varley A, Warren FC, Richards SH (2016). The effect of nurses’ preparedness and nurse practitioner status on triage call management in primary care: a secondary analysis of cross-sectional data from the ESTEEM trial. Int J Nurs Stud.

[92] Warren FC, Calitri R, Fletcher E (2015). Exploring demographic and lifestyle associations with patient experience following telephone triage by a primary care doctor or nurse: secondary analyses from a cluster randomised controlled trial. BMJ Qual Saf.

[93] Salisbury C, Goodall S, Montgomery AA (2007). Does advanced access improve access to primary health cam? Questionnaire survey of patients. Br J Gen Pract.

[94] Pope C, Banks J, Salisbury C, Lattimer V (2008). Improving access to primary care: eight case studies of introducing Advanced Access in England. J Health Serv Res Policy.

[95] Lopez Segui F, Vidal-Alaball J, Sagarra Castro M (2020). General Practitioners’ perceptions of whether teleconsultations reduce the number of face-to-face visits in the Catalan public primary care system: retrospective cross-sectional study. J Med Internet Res.

[96] Siddiqui F, Sidhu B, Tahir MA (2017). Using ‘active signposting’ to streamline general practitioner workload in two London-based practices. BMJ Open Qual.

[97] Fagerlund AJ, Holm IM, Zanaboni P (2019). General practitioners’ perceptions towards the use of digital health services for citizens in primary care: a qualitative interview study. BMJ Open.

[98] Youde K (2010). Broadband: the first decade. The Independent.

[99] Baker N (2024). UK mobile phone statistics, 2023. https://www.uswitch.com/mobiles/studies/mobile-statistics.

[100] Ofcom (2022). Mobile networks and spectrum: meeting future demand for mobile data.

[101] British Medical Association, NHS England (2019). Investment and evolution: a five-year framework for GP contract reform to implement The NHS Long Term Plan.

[102] Zhao P, Yoo I, Lavoie J (2017). Web-based medical appointment systems: a systematic review. J Med Internet Res.

[103] Salisbury C, Murphy M, Duncan P (2020). The impact of digital-first consultations on workload in general practice: modeling study. J Med Internet Res.

[104] Department of Health (2002). Achieving and sustaining improved access to primary care. https://webarchive.nationalarchives.gov.uk/ukgwa/20070305164919/.

[105] Government HM (2000). The NHS Plan.

[106] Government HM (2011). Equality Act 2010. Section 149. https://www.legislation.gov.uk/ukpga/2010/15/section/149.

